# An expert-based job exposure matrix for large scale epidemiologic studies of primary hip and knee osteoarthritis: The Lower Body JEM

**DOI:** 10.1186/1471-2474-15-204

**Published:** 2014-06-13

**Authors:** Tine Steen Rubak, Susanne Wulff Svendsen, Johan Hviid Andersen, Jens Peder Lind Haahr, Ann Kryger, Lone Donbæk Jensen, Poul Frost

**Affiliations:** 1 Department of Occupational Medicine, Slagelse Hospital, Ingemannsvej 18, 4200 Slagelse, Denmark; 2 Danish Ramazzini Centre, Department of Occupational Medicine, Regional Hospital West Jutland - University Research Clinic, Gl. Landevej 61, 7400 Herning, Denmark; 3 Department of Occupational and Environmental Medicine, Copenhagen University Hospital, Bispebjerg, 2200 Copenhagen NV, Denmark; 4 Danish Ramazzini Centre, Department of Occupational Medicine, Aarhus University Hospital, Aarhus, Denmark

## Abstract

**Background:**

When conducting large scale epidemiologic studies, it is a challenge to obtain quantitative exposure estimates, which do not rely on self-report where estimates may be influenced by symptoms and knowledge of disease status. In this study we developed a job exposure matrix (JEM) for use in population studies of the work-relatedness of hip and knee osteoarthritis.

**Methods:**

Based on all 2227 occupational titles in the Danish version of the International Standard Classification of Occupations (D-ISCO 88), we constructed 121 job groups comprising occupational titles with expected homogeneous exposure patterns in addition to a minimally exposed job group, which was not included in the JEM. The job groups were allocated the mean value of five experts’ ratings of daily duration (hours/day) of standing/walking, kneeling/squatting, and whole-body vibration as well as total load lifted (kg/day), and frequency of lifting loads weighing ≥20 kg (times/day). Weighted kappa statistics were used to evaluate inter-rater agreement on rankings of the job groups for four of these exposures (whole-body vibration could not be evaluated due to few exposed job groups). Two external experts checked the face validity of the rankings of the mean values.

**Results:**

A JEM was constructed and English ISCO codes were provided where possible. The experts’ ratings showed fair to moderate agreement with respect to rankings of the job groups (mean weighted kappa values between 0.36 and 0.49). The external experts agreed on 586 of the 605 rankings.

**Conclusion:**

The Lower Body JEM based on experts’ ratings was established. Experts agreed on rankings of the job groups, and rankings based on mean values were in accordance with the opinion of external experts.

## Background

Primary hip and knee osteoarthritis (OA) are common musculoskeletal disorders, not just in older age groups, but also in the working age population [1,2]. These disorders constitute the main indications for total joint replacement surgery. Recent reviews have concluded that there is evidence of a causal relationship between occupational mechanical exposures and primary hip and knee OA, although important limitations still exist, particularly due to modest quality of exposure assessment [3-8]. For primary hip and knee OA quantitative exposure-response relationships for specific exposures remain to be established [3,4].

Self-reported mechanical exposures have unique advantages [9] and have been used also in recent studies of hip and knee OA [10,11]. However, self-reported exposures entail validity problems to the extent that individuals with symptoms – or knowledge of disease status even in the absence of symptoms – overestimate their exposures leading to inflated estimates of exposure-response relationships. This source of bias is of major concern in cross-sectional and case – control studies of symptomatic OA and may also be a problem in prospective longitudinal studies because patients may have endured gradually increasing joint symptoms for several years before they are diagnosed with primary hip or knee OA. Thus, the evidence-base for a causal relationship between symptomatic primary hip and knee OA and occupational mechanical exposures would be enhanced by studies (preferably longitudinal) using quantitative measures of generic exposures that are assessed independently of the musculoskeletal symptom status of the participants.

In general population studies, systematic observations and direct technical measurements are resource demanding even if the methods are only applied to small subsets of the study population, and relevant equipment may not exist. To our knowledge, these methods have not been used in studies on primary hip OA. Observations in a few selected occupations have been used in studies of knee OA defined radiographically (irrespective of symptoms) [12-14], and direct technical measurements have been used in a study comparing floor-layers and carpenters with respect to primary knee OA defined clinically and radiographically [15].

Retrospective exposure assessment is a special challenge [16] and for this purpose expert ratings may be the best method available [17]. Expert ratings may be used either on a case-by-case basis [18] or as a means of constructing a job exposure matrix (JEM) [17]. JEMs have proved valuable in occupational epidemiology [19-22], but mechanical exposures have rarely been included. We are aware of one general population expert based JEM focussing on exposures to the lower extremities. The JEM was based on consensus expert ratings of proportions of the working day involving six mechanical exposures [23]. The researchers were restricted by the fact that the 40 job groups were fixed entities developed for other purposes. This meant that the job groups were often inhomogeneous as regards mechanical exposures to the lower limbs, e.g., one of the groups contained both writers and athletes. Hence, some of the jobs were grouped in a way that would obscure their impact [17,24]. An ambitious Finnish general population JEM covered mechanical exposures that were ranked by experts (0–1 or 0–2) [25], but the JEM did not provide quantitative estimates that could be used to establish thresholds for hazardous generic exposures. This was also the case in two recent studies that constructed JEMs for assessment of the likely frequency of exposure to certain work activities like heavy lifting and kneeling within job groups [26,27]. The studies used information from earlier surveys [26] or studies [27] to estimate the likely exposure frequencies. The United States’ Department of Labour has also assessed mechanical exposures in the O*Net Analyst Database http://www.onetcenter.org/database.html. For upper limb exposures, a general population shoulder JEM has been established based on experts’ ratings [28] and the first steps have been taken to construct general population JEMs based on direct technical measurements [29,30]. In this paper we present a new two-dimensional JEM with job groups based on all currently used occupational titles in the Danish version of the International Standard Classification of Occupations (D-ISCO 88) [31] on one axis and expert ratings of five specific mechanical exposures to the lower extremities on the other.

Our aim was to provide independent exposure estimates for use in general population studies of the influence of mechanical exposures to the lower body on risk of primary hip and knee OA leading to total joint replacement [32]. We focussed on standing/walking [33,34], kneeling/squatting [12], total load lifted per day [3,4], and daily frequency of lifting loads weighing ≥20 kg [35]. We also assessed whole-body vibration even though previous studies have not shown associations [36,37].

## Methods

### Screening of occupational titles

As our starting point, we took the total list of 2227 different occupational titles that are covered by 372 D-ISCO codes in D-ISCO 88 [31]. D-ISCO 88 is slightly different from the international version - some English occupational titles have no counterpart in the Danish version and vice versa. It is worth noting that some occupational titles have different codes in the two versions, for instance, “furniture mover” has code 9330 in D-ISCO 88 and 9333 in the international version, and “cutter, fish” has code 8271 in D-ISCO 88 and 7411 in the international version. We have provided our JEM with international occupational titles and classification codes, where possible.

We screened the list of occupational titles to exclude obsolete or very rare titles and to identify occupations with minimal exposures to the lower limbs. To ensure that few occupational titles with low exposures would be incorrectly classified into a high exposure category (i.e. to ensure a high specificity) [38], we decided that to be considered more than minimally exposed, at least one of the following had to be present in the job: standing/walking ≥6 hours/day, kneeling/squatting > ½ hour/day [34], whole body vibration >2 hours/day [39], lifting >2 tons/day, or lifting loads weighing ≥20 kg ≥10 times/day. We considered driving tractors and heavy machinery (e.g. road rollers, excavators, bulldozers, and trench-digging machines) to entail whole-body vibration as opposed to riding cars, lorries, trucks, and trains.

### Establishing job groups with expected homogeneous exposure patterns

Exposed occupational titles were collapsed into job groups with expected homogeneous exposure patterns regarding all exposures that we intended to assess [40]. Thus, two occupational titles were classified in different job groups if they differed with respect to just one exposure among the five. D-ISCO 88 groups were split up if their exposures were judged to differ, e.g. we classified “barkeeper” and “general manager, camping site” (both coded 1315 in D-ISCO 88) in different job groups. On the other hand, different D-ISCO 88 codes could be categorised in the same job group.

### Expert ratings

Five experts rated the exposures - four occupational health physicians, who all had at least 10 years of experience from departments of occupational medicine (SWS, PF, JHA, and JPH), and a medical graduate specialising in occupational medicine (TR). The number of experts was chosen in accordance with recent recommendations [41]. The grouping of occupational titles was discussed among the experts, and any disagreements were settled in consensus. All experts participated in a pilot rating of ten randomly selected job groups, which did not lead to any adjustments of the rating process. Each expert independently entered his/her ratings into an electronic database. For each job group, the experts were asked to rate the mean number of hours per day spent standing/walking, kneeling/squatting, and exposed to whole-body vibration (in half-hour intervals). Sitting was also assessed so that the experts could ensure that standing/walking, kneeling/squatting, and sitting added up to a full working day defined as eight hours. For lifting, the experts were asked to estimate the total load lifted (kg/day) and the frequency of lifting loads weighing ≥20 kg (times/day). The ratings were compared and gross outliers were discussed at a panel meeting. Most disagreements arose due to misinterpretation of occupational titles and components of the jobs. After reaching a consensus on job components, a number of outlying estimates were changed by each expert, and two job groups were re-evaluated by all five experts. For each job group, the means of the experts’ final ratings were included in the JEM. In this way, we aimed to synthesize the best features of panel team work/consensus ratings and independent assessments [23,41,42].

### Inter-rater agreement

We used weighted kappa statistics to evaluate agreement between each possible pair-wise combination of the five raters, i.e. 10 kappa estimates per exposure. For each rater and each exposure, the 121 exposed job groups were ranked and then divided into five groups of increasing exposure (four groups with 24 job groups in each and one with 25 job groups). We then evaluated agreement of rankings using the wording suggested by Landis & Koch [43]. Whole-body vibration was not evaluated due to few exposed job groups.

### Face validity

To validate the JEM in the absence of a gold standard, we ranked the job groups according to their mean values for each exposure variable. For standing/walking the categories were 0- < 2, 2- < 4, 4- < 6, 6+ hours/day, for kneeling/squatting and whole-body vibration 0, 0- < ½, ½- < 1, 1- < 2, 2- < 4, 4+ hours/day, for total load lifted per day: 0- < 500, 500- < 1000, 1000- < 2000, 2000- < 4000, 4000+ kg/day, and for daily frequency of lifting loads weighing ≥20 kg 0- < 5, 5- < 10, 10- < 20, 20+ times/day. Two experts (AK and LDJ), who were not involved in the expert ratings, stated if they agreed with the rankings and suggested adjustments.

## Results

We excluded 117 occupational titles that we considered rare or obsolete, and we initially judged 1421 occupational titles to be minimally exposed (e.g. teachers, office workers, physicians, police- and firemen). This left 689 occupational titles – representing 168 D-ISCO 88 codes - that were divided into 121 job groups, each containing 1–34 different occupational titles. Of the 689 Danish occupational titles, 465 had a counterpart in the international version of ISCO (including some with different codes in the two versions of the classification). A total of 91 occupational titles could be translated into English, but the English translations were not represented in the international version. We were not able to translate the remaining 133 Danish occupational titles into English. Hence, the English version of the JEM contains 556 occupational titles and 157 ISCO codes.

**Figure 1:**
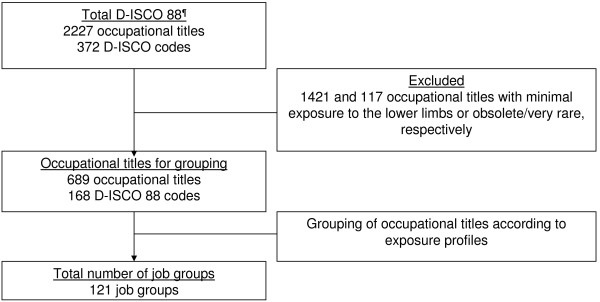
**The flow of occupational titles and related D-ISCO 88 codes to create the final job groups that where included in the Lower Body Job Exposure Matrix.**^¶^D-ISCO 88 – Danish version og the International Standard Classification of Occupations

The flow from total D-ISCO 88 to final number of job groups is shown in Figure 1. Grand means and exposure percentiles in the JEM are shown in Table 1. The two job groups with the highest exposure to standing/walking were workers in the fish-processing industry and slaughterhouse workers. For kneeling/squatting, the two groups with the highest exposures were floor-layers and paviours. For whole-body vibration, tractor drivers, drivers of heavy machinery, and workers in quarries had the highest exposures. Loaders in airports and scaffolders were assessed to be most highly exposed to heavy lifting, both regarding total load lifted per day and daily frequency of lifting loads weighing ≥20 kg.

**Table 1:** Distribution of exposures across 121 job groups comprising 686 occupational titles in The Lower Body Job Exposure Matrix

**Exposure**	**Grand mean**	**10**^**th**^**percentile**	**50**^**th**^**percentile**	**90**^**th**^**percentile**
Standing/walking (hours/day)	5.3	3.0	5.7	6.6
Kneeling/squatting (hours/day)	0.4	0	0.2	1.1
Whole-body vibration (hours/day)	0.1	0	0	0.2
Total load lifted (kg/day)	955	193	590	2525
Frequency of lifting loads weighing ≥20 kg (times/day)	10.2	1.2	6.0	21.5

The table excludes 1421 occupational titles estimated to entail mechanical exposures below the screening levels* for all five exposures.

*Standing/walking ≥6 hours/day, kneeling/squatting > ½ hour/day, whole-body vibration >2 hours/day, lifting >2 tons/day, or lifting loads weighing ≥20 kg ≥10 times/day.

To illustrate the composition of the JEM, three job groups are shown in Table 2. Since occupational titles were only left out if their exposures were estimated to be below the screening levels for all five exposure variables, some of the job groups in the JEM turned out to be less exposed than the initial cut-off values used to identify occupational titles with minimal exposures, cf. the 10^th^ percentiles in Table 1.

**Table 2:** Three job groups from The Lower Body Job Exposure Matrix

**Job group number**	Danish industry number*	**ISCO**^**†**^	**Occupational titles**	**Exposure estimates (mean of five experts’ ratings) for a working day of eight hours**				
				**Standing/walking (hours/day)**	**Kneeling/squatting (hours/day)**	**Whole-body vibration (hours/day)**	**Total load lifted (kg/day)**	**Frequency of lifting loads weighing ≥20 kg (times/day)**
1	1	1311	General manager, agriculture (except those in nurseries and green houses)	4.8	0.2	1.1	900	9.6
		6111	Farm worker, skilled/potato					
		6112	Farmer, fruit					
		6121	Farmer, horse breeding; farm worker					
		6122	Farmer, poultry/hatching and breeding; breeder, poultry					
		6129	Farmer, fur/non-domesticated animals					
		6130	Farmer, mixed farming; farm worker, skilled/mixed farming					
		9211	Groom, stud; labourer, farm					
		no ISCO	Farmer, fur/non-domesticated animals (mink); groom					
12	2	8231	Machine-operator, rubber; machine-operator, vulcanising/rubber goods	6.7	0.2	0.0	1150	6.4
48	3	8161	Operator, generator/electric power	4.0	0.2	0.0	380	2.4
		8162	Operator, boiler plant/steam					

*Numbers according to the 9-grouping of industries defined by Statistics Denmark: 1 – Agriculture, fishing and quarrying, 2 – Manufacturing, 3 – Electricity, gas and water supply.

^†^ISCO codes in accordance with the International Standard Classification of Occupations.

Table 3 shows inter-rater agreements for each of four exposures. Kappa values were on average lowest for kneeling/squatting (mean weighted kappa = 0.36) and highest for total load lifted per day (mean weighted kappa = 0.49). In general, agreements were fair to moderate.

**Table 3:** **Inter-rater agreement of the ranking of four exposures (grouped into quintiles) and level of agreement**^**a**^

**Exposure**	**Weighted kappa**			**Level of agreement**		
	**Mean**	**Min**	**Max**	**Fair (number)**	**Moderate (number)**	**Substantial (number)**
Standing/walking	0.41	0.34	0.52	6	4	0
Kneeling/squatting	0.36	0.30	0.41	8	2	0
Total load lifted	0.49	0.34	0.63	1	8	1
Frequency of lifting loads weighing ≥20 kg	0.38	0.29	0.43	7	3	0

^a)^Poor (below 0.0), slight (0.00-0.20), fair (0.21-0.40), moderate (0.41-0.60), substantial (0.61-0.80), almost perfect (0.81-1.00) [40].

Ten pair-wise comparisons between five raters for each exposure.

Two external experts checked the ranking of job groups, and agreed with 586 out of the 605 original ratings (5 exposure variables for each of 121 job groups). One of the experts suggested 10 changes (seven increases and three decreases of exposure), and the other suggested nine changes (four increases and five decreases). None of these suggestions were the same. Accordingly, we did not change the JEM.

## Discussion

We presented the development of a JEM cross-classifying 121 job groups with five generic mechanical exposures to the lower extremities. The JEM encompasses the whole labour market in Denmark and provides quantitative exposure measures, except for a minimally exposed job group, which was not included in the JEM. In general, the agreement between the experts’ rankings was fair to moderate and the face validity was found to be high.

The inter-rater agreements were higher than the mean weighted kappa values of between 0.2 and 0.3, which have been previously reported for standing, heavy lifting, and kneeling [23]. This reflected the fact that we did not have the constraints faced by the authors of the just-mentioned study with respect to the grouping of occupational titles, and the fact that their comparisons were based on initial ratings, whereas our comparisons were made after correction of outlying estimates. We estimated that floor-layers were exposed to kneeling/squatting for on average 3.5 hours/day, which is comparable to estimates based on observations and measurements [13-15,44]. It seems reasonable that no job groups obtained a higher mean for this exposure. We are not aware of other comparable exposure estimates based on observations and measurements. Our first priority was to rank the job groups in a valid way since this is a precondition for exploring exposure-response relationships. The face validity was high, so we think that our quantitative estimates reflected the ranking of true exposures quite well, whereas the absolute values are more questionable.

We grouped occupational titles instead of D-ISCO codes because D-ISCO codes are based on skills required to fulfil tasks and duties of the jobs and thus may not reflect specific exposures. Maybe the agreement could have been improved if we had provided the experts with brief texts describing the work content of the occupational titles represented in the job groups [41]. Such descriptive texts could also make it easier to adapt the matrix for studies of other populations. We refrained from the use of exposure vignettes because our exposure assessment panel included experienced specialists, who knew the tasks of the majority of occupational titles present in the job groups. Another way of obtaining better agreement could be to use 10–15 benchmarks in terms of occupational titles representing specific job groups, which the experts consensus rated before the remaining rating process. In this way the experts could calibrate their estimates to a common scale [17,28,45].

We designed the job axis of The Lower Body JEM to contain as homogenous exposure groups as possible [40]. Based on theories of classic and Berkson errors, group-based exposure assessment should be less subject to attenuation bias than individual-based approaches [38]. However, to the extent that we mixed occupational titles with high and low true exposures within the job groups, the observed mean values of the job groups would erroneously seem similar (ultimately, we could have constructed our job groups in such a poor way that all group means were equal, meaning that we would be unable to detect exposure-response relationships). Subsequently, quantitative exposure-response relationships obtained by the JEM may be calibrated by validation studies based on observation or technical measurements of selected exposures for selected occupational titles or job groups.

We classified more than 50% of all occupational titles as minimally exposed. To the extent that these titles were in fact more than zero-exposed, exposure-response relationships based on the JEM would underestimate true associations (if the possibility of a U-shaped relationship is disregarded). The omission of occupational titles judged to entail minimal exposures precludes the use of the JEM to study effects of these exposures, which may be relevant with respect to other outcomes than OA [46]. As a future refinement of the JEM, the large group of minimally exposed occupational titles may be subdivided and provided with exposure estimates. Some of the job groups in the JEM received one or more exposure estimates that were lower than the cut-off points used in the screening process. We kept these estimates in the JEM to reduce the risk of underestimation of associations due to misclassification of exposures that were not minimal.

The use of probability of exposure has been proposed as a means to minimize bias due to misclassification of exposures [47], and has been used in recent studies of lower body exposure [26,27]. However, the probability approach may be more meaningful in studies of chemical exposures that occur in specific occupational groups, where some group members are exposed and others are not. For mechanical exposures, the situation is typically different. For instance, standing/walking is widely distributed and does not occur in an on-or-off manner, and exposure to whole-body vibration occurs in few occupations where the majority of the group members are exposed to some extent. Therefore, we found it more informative to provide quantitative estimates of mean exposures.

We did not use different estimates for men and women within the same occupation. Women in heavily exposed jobs may actually be less exposed than their male colleagues, for instance due to gender segregation of tasks within jobs. This would have the effect that women would erroneously seem to be less affected by heavy exposures than men. A perspective for improvement of the JEM could be to provide gender specific estimates for selected groups [26]. However, the Danish labour market is to a large extent gender segregated so that men and women work in different jobs, which means that the practical significance of such an effort may be limited.

The job groups were constructed to have similar exposure profiles across the five exposure variables that we assessed. The relatively large number of job groups means that it will be possible to update specific exposure estimates in The Lower Body JEM as new knowledge is obtained, and other researchers will be able to modify the JEM for use in different study populations. The JEM has already proved useful in a study of the work-relatedness of inguinal hernias [48] and in our recent case–control study of hip OA [32]. In these studies, the exposure estimates from the Lower Body JEM were used to calculate cumulative exposure measures. The JEM may also prove useful for research into e.g. varicose veins, where prolonged sitting or standing/walking have been suggested as risk factors [49].

When the JEM is applied for exposure assessment in an epidemiologic study, a high prevalence of job groups with high inter-rater agreement and a low prevalence of job groups with low inter-rater agreement would yield kappa values for the ranking of exposures in the study population, which are larger than calculated for the JEM per se (this situation seems quite likely since large job groups would be particularly well known to the experts). In this situation, the JEM-based exposure assessment must be expected to lead to risk estimates that are closer to the real than suggested by the presented kappa values, provided that high agreement reflects a better estimate of the true exposure. Thus, the influence of agreement between raters on the probability of biased risk estimates is related to the prevalence of the job groups in the study population. It may even be a design option to restrict study populations to job groups with relatively high agreement between raters to counteract biased risk estimates.

Until more accurate and precise methods for exposure assessment have been developed that are feasible for use in large scale population studies of hip and knee OA and other lower extremity disorders, we find it promising to explore the avenue of a JEM approach based on expert ratings of mechanical exposures.

## Conclusion

We have developed a JEM for use in general population studies of primary hip and knee OA with a potential for use in studies of other health outcomes and in other countries with working conditions and industry compositions similar to the Danish. We do not see the matrix as a fixed entity, but an entity to be developed and updated, when more knowledge becomes available.

## Abbreviations

JEM: Job exposure matrix; ISCO: International Standard Classification of Occupations; D-ISCO: Danish version of International Standard Classification of Occupations; OA: Osteoarthritis.

## Competing interests

None of the authors have any competing interest concerning this study.

## Authors’ contributions

All authors were involved in conception and design of the study. TR constructed the template for the JEM. SWS, JPH, JHA, PF, and TR rated the exposures, and AK and LDJ carried out the face validity assessment. TR and PF analysed the data. TR and SWS drafted the paper. All authors revised paper drafts critically for important intellectual content and approved the final version.

## Pre-publication history

The pre-publication history for this paper can be accessed here:

http://www.biomedcentral.com/1471-2474/15/204/prepub
